# High glucose-induced inhibition of osteoblast like *MC3T3-E1* differentiation promotes mitochondrial perturbations

**DOI:** 10.1371/journal.pone.0270001

**Published:** 2022-06-17

**Authors:** Claudia Medeiros, Joseph M. Wallace

**Affiliations:** Department of Biomedical Engineering, Indiana University–Purdue Indianapolis (IUPUI), Indianapolis, Indiana, United States of America; Kyungpook National University School of Medicine, REPUBLIC OF KOREA

## Abstract

Diabetes mellitus is a metabolic disorder that causes health concerns worldwide. Patients with diabetes exhibit multisystemic symptoms, including loss of bone quality over time. The progressive deterioration of bone promotes failure to withstand damage and increases the risk of fractures. Much of the molecular and metabolic mechanism(s) in diabetic bone remains unclear. *In vitro* studies suggest that hyperglycemia inhibits mineralization, affecting bone formation and function. In this study, inhibition of osteoblast differentiation was induced using hyperglycemia to assess whether high glucose promotes mitochondrial impairment along with altered bone matrix formation. It was hypothesized that bone energy metabolism would be altered in these cells as calcium deposition, a key phase for bone function, is suppressed. Early passages of osteoblast like *MC3T3-E1* cells were differentiated under normal and high glucose conditions. To investigate osteoblast differentiation, we quantified calcium accumulation by alizarin red staining and analyzed immunoblots of key proteins. To assess mitochondrial function, we quantified mitochondrial DNA (mtDNA), detected expression and function of key proteins from the Tricarboxylic (TCA) cycle, measured mitochondrial respiration, and fuel oxidation of alternative nutrients. Results confirmed previous work showing that mineralization was inhibited and AKT expression was reduced in high glucose-treated bone cells. Unexpectedly, high glucose-treated osteoblast cells utilize both mitochondrial respiration and glycolysis to maintain energy demands with partial help of fatty acid for reliance of baseline bioenergetics. These metabolic shifts suggest that hyperglycemia maintain bone metabolic needs in an early differentiated state concurrent to the inhibition in bone matrix formation.

## 1. Introduction

Diabetes mellitus (DM) is public health problem that affects over 463 million people worldwide [1]. There are two types of DM: type 1 diabetes (T1D) and type 2 diabetes (T2D). In T1D, which is usually detected at a young age, the immune system attacks the pancreatic β cells and patients produce little or no insulin [2]. T2D has a higher prevalence in the patient population. Although the β cells produce insulin, cells in the body become insensitive likely driven by chronically elevated glucose in the blood. As the β cells deploy even more insulin to overcome this insensitivity, they eventually wear out and insulin levels in the blood drop [2]. Diabetes is a metabolic disorder with multisystemic complications contributing to cardiovascular disease, retinopathy, and chronic kidney disease [3]. In addition, DM patients have an increase in bone fracture risk [4, 5]. In these patients, there is deterioration of bone over time with loss of bone quality due to pathophysiological factors, including hyperglycemia, insulin resistance, and oxidative stress driven by the accumulation of advanced glycation end products (AGE) [6, 7]. In diabetic animals, bone integrity is compromised as well with several factors that negatively impact cell processes (bone formation and remodeling) [8] that give rise to abnormal bone microstructure and promote accumulation of AGEs in the bone matrix [9].

Both diabetes and bone disease are multisystemic disorders that exhibit metabolic alterations. To gain insight into metabolic impairment, research efforts have accelerated in an effort to understand mitochondrial function. Mitochondrion is the organelle that supports the cell to perform several roles including ATP synthesis and Oxidative Phosphorylation (OXPHOS). In DM patients, mitochondrial dysfunction is characterized by a reduction of OXPHOS, ATP generation, as well as a decrease of mitochondrial biogenesis biomarkers [10], reduction of mitochondrial content, and increased oxidative stress [11]. In human diabetic pancreatic β cells, expression of electron transport chain (ETC) proteins were elevated with a reduction in ATP levels [12]. In β cells derived from the MKR mouse model, there was a decrease in mitochondrial respiration, mitochondrial content, and accumulation of ROS [13]. In human diabetic skeletal muscle, reduced activity of both NADH:O2 oxidoreductase and citrate synthase (CS) was observed [14] along with reduced Cytochrome c oxidase content [15] and decreased OXPHOS gene expression [16].

Bone is a dynamic tissue that can withstand damage and is involved in various roles including mineral metabolism. Osteoblasts are responsible for the synthesis of bone matrix and mineral deposition [17]. In T2D, several factors contribute to osteoblast dysfunction leading to bone quality degradation [4]. To help elucidate T2D in bone cells, high glucose has been used to mimic the disease *in vitro*. Previous work showed that high glucose inhibits mineralization in MC3T3-E1 osteoblast-like cells [18, 19].

Healthy osteoblasts utilize glucose during differentiation via both oxidative phosphorylation and aerobic glycolysis with a preference for the latter [20]. In glucose metabolism, pyruvate is a by-product of glucose breakdown that can be used in glycolysis [21]. Alternatively, pyruvate can be used to initiate the tricarboxylic acid (TCA) cycle as a source of Acetyl-CoA [22]. The TCA cycle is the central metabolic pathway of mitochondria to generate intermediate metabolites for other metabolic pathways like fatty acid (FA) synthesis. FA oxidation also generates Acetyl-CoA and, hence, the main intermediate to bridge carbohydrate and lipid metabolisms [23]. FA metabolism is important to yield energy necessary for bone formation [24].

Early studies suggested a smaller role of the TCA cycle to oxidize glucose in bone [25, 26]. However, recent work identified elevated levels of intermediate metabolites from the TCA cycle along with glycolysis and gluconeogenesis in diabetic patients [27]. Since the TCA cycle is affected in DM, it is plausible to consider dysregulation of FA and/or other fuels in diabetic bone. These observations suggest that DM exhibits changes in the bioenergetics state of various metabolic pathways, and mechanistic insight into osteoblast differentiation in this disease state may have implications to mitochondrial dysfunction. To bridge the gap between T2D and bone disorders, we aim to determine expression and function of key TCA enzymes, cellular respiration, and fuel oxidation. In this report, we describe the results of a high glucose treatment of osteoblastic cells designed to mimic T2D *in vitro* and identify altered bone and metabolic defects. From this treatment, high glucose inhibited osteoblast mineralization and revealed that hyperglycemic conditions in differentiated osteoblast cells leads to metabolic shifts and mitochondrial impairment.

## 2. Material and methods

### 2.1 Cell culture

MC3T3-E1 Subclone 4 (ATCC® CRL-2593) murine pre-osteoblasts were obtained from the American Type Culture Collection (ATCC, Manassas, VA). Cells were cultured in either minimal growth media (GM) or osteogenic media (OM) for differentiation, as previously described [28] with the following modifications for OM: final concentrations at 1.46mM of β-Glycerophosphate (BGP) and 50 μg/mL ascorbic acid 2-phosphate (Sigma Aldrich, St. Louis, MO) at 37°C. Cells were treated with osteogenic media (OM) in the presence of low glucose (5.5mM) or supplemented with 25 mM of high glucose (HG) at a final concentration of 30.5mM to reflect glucose serum levels of approximately 100 and 540 mg/dl, respectively. Cells were seeded into 6 well-plates (10,000 cells per well). Once cells reached 90% confluence, differentiation was performed for three or four weeks. Experiments were performed on cells under normoxic conditions at passage four (p#4) from the initial passage obtained from ATCC®. All experiments were performed as N = 6 independent experiments in triplicates or otherwise stated in the respective figure legend.

### 2.2 Alizarin Red S staining

Alizarin Red S kit and protocol were obtained from ScienCell™ Research laboratories and were used to detect calcium deposits with the following modification: cold 70% ethanol was used for 1h in the fixative step. Cells were cultured for four weeks under the following conditions: α -minimal growth supplemented media with low glucose (GM), osteogenic media with low glucose (OM), α -minimal growth supplemented media with high glucose (GM, HG+), osteogenic media with high glucose (OM, HG+). A total of six biological samples were collected per condition per week.

### 2.3 DNA extraction

Genomic DNA (gDNA) was extracted from differentiated samples treated with HG for four weeks using Isolate II Genomic DNA kit (Bioline, USA). Both control and treated DNA samples were extracted at the end of each week for four weeks of treatment. A total of six biological samples per condition were collected weekly.

### 2.4 Quantitative Polymerase Chain Reaction (qPCR) to quantify mitochondrial DNA (mtDNA) content

Relative copy number of mtDNA in differentiated osteoblasts was analyzed using real-time qPCR methods as described [29]. Two amplicons were amplified to represent nuclear DNA (nDNA) and mitochondrial DNA (mtDNA). nDNA amplicon used as normalizer was the beta 2 microglobulin gene (*B2M*) and was amplified using forward primer 5’ TGATGGTGAGGTCTGGAATG 3’ and reverse primer 5’ GCAGGTTCAAATGAATCTTCAG’, giving a fragment of 106 bp. The mtDNA amplicon used was *ND1* region of mouse mitochondrial mtDNA and was amplified using 5’ TCTAATCGCCATAGCCTTCC 3’ and reverse primer 5’ GGTTGTTAAAGGGCGTATTGG 3’, giving a fragment of 160 bp. qPCR analysis was performed using a QuantStudio 3 Real-Time PCR systems (Thermo Fisher) and amplicons were detected with SYBR Green. The mtDNA content (mtDNA/B2M ratio) was calculated using the formula: mtDNA content = 1/2^ΔCt^, where ΔCt = Ct^mtDNA _^ Ct^B2M^.

### 2.5 Western blot

Whole cell lysates were prepared in RIPA lysis buffer (Thermo Scientific™) with protease inhibitors after three and four weeks of differentiation and high glucose treatment. After centrifugation of the lysate (10000g, 4 min), soluble proteins were isolated in the supernatant, and protein concentration was determined according to the BCA method. Samples were denatured with equal volume of 2x SDS-PAGE sample loading buffer prepared following Bio-rad specifications. A total of 20 μg of whole lysate samples were separated on a 10% SDS-polyacrylamide mini-gel. Proteins were transferred electrophoretically to polyvinylidine difluoride (PVDF) membranes for 90 min at 100V. Membranes were cut in strips according to the molecular weight of proteins examined prior to primary antibody incubation. Membranes were blocked overnight in 5% milk-PBS and incubated overnight with primary antibodies at the following concentrations: α-AKT (1:2,000) and α-RUNX2 (1:2,000) from Cell Signaling Technology; α-β-CATENIN (1:10,000), α-SUCLA2 (1:10,000), α-SUCLG2 (1:5,000), and α-SUCLG1 (1:5,000) from GeneTex; α-CS (1:5,000) and α-ACO2 (1:10,000) and CS (1:5,000) from Abcam. α-β-ACTIN (1:2,500) from Sigma was used as a loading control for normalization. Membranes were washed three times with PBS-0.05% Tween 20 prior to imaging. The secondary antibody was detected using the chemiluminescent HRP substrate (ECL) by Millipore (USA). Membranes were documented with the ChemiDocMP Imaging System (Bio-Rad).

### 2.6 Succinyl—CoA (SCS) enzyme activity

Protein lysates were extracted after 3 weeks of differentiation and HG treatment with Cultured Cell Sonication Medium pH to 7.2 buffer (25mM KPO4 monobasic, 2mM HEPES, and 5mM MgCl2). SCS activity was measured at 30°C in whole cell lysates (5μg) from differentiated osteoblasts in the direction of succinate to Succinyl–CoA reaction as previously described [30] with the following modifications: SCS buffer final volume of 175μL containing 50 mM potassium phosphate, pH 7.2, 10 mM MgCl_2_, 0.2 mM succinyl-CoA, 2 mM ADP (for A-SCS) or 2 mM GDP (for G-SCS), and 0.2 mM DTNB. Reactions were initiated by adding Succinyl-CoA wand DTNB in quick succession. Rates were corrected by subtracting ADP- and GDP- in the absence of both dNDPs. The interaction of CoA-SH group (released from Succinyl- CoA) with DTNB generated thionitrobenzoate that was measured spectrophotometrically at 412 nm using Spectra® M5 plate reader (Molecular Devices). A total of six biological samples per condition (ADP, GDP or No ADP/GDP) in triplicates were used to determine SCS activity All activities were calculated as nmoles/minute/mg protein and expressed as percentage of either OM (control) or OM, HG+ activity.

### 2.7 Citrate synthase enzyme activity

Protein lysates were extracted after 3 weeks of differentiation and HG treatment with Culture Cell Sonification Medium described in section 2.6. Citrate synthase (CS) activity was measured by the reduction of 5,5’-dithiobis (2-nitrobenzoic acid) (DTNB) that was coupled to the reduction of acetyl-CoA by CS in the presence of oxaloacetate (OAA). The reaction was measured spectrophotometrically at 412 nm using SpectraMax® M5 (Molecular Devices). The reaction mixture was prepared as described previously [31]. A total of six biological samples per condition were used to determine CS activity. All activities were calculated as nmoles/min/mg protein. Data correspond to two technical replicates of N = 6 independent experiments in triplicates.

### 2.8 Cellular respiration assay

#### 2.8.1 Mito stress test

Agilent Seahorse XF HS mini extracellular flux analyzer was used to measure the rates of oxygen consumption in differentiated osteoblasts. Cells were plated on XF HS mini cell culture microplates at a density of 5,000 cells per well. Differentiation initiated the following day and lasted three weeks prior the assay. XF HS mini cartridge was equilibrated with the calibration solution overnight at 37° C. XF HS mini assay media supplemented with 10mM glucose, 1 mM Pyruvate, and 2mM of L-glutamine in DMEM (Dulbecco’s Modified Eagle Medium) was prepared and pH adjusted to 7.4 on the day of the experiment. XF assay media was used to prepare cellular stress reagents to provide the following final concentrations: 2μM Oligomycin, 2μM FCCP, 0.5μM Antimycin A and 0.5μM Rotenone. All the reagents were loaded in the ports as suggested by Agilent Technologies. Oxygen consumption rate (OCR) for mitochondrial respiration/OXPHOS and extracellular acidification rate (ECAR) glycolysis were measured for 3 min with 3 min of mixing and 2 min of waiting period. A total of 12 measurements were taken. OCR and ECAR rates were normalized by cell number and expressed as nmoles oxygen/min/1000 cells. All fundamental parameters of mitochondrial respiration were calculated using Agilent Seahorse Wave Controller software.

#### 2.8.2 Mito fuel flex test

To determine if other nutrients are utilized for fuel oxidation, Fuel Dependency was tested in cells cultured in osteogenic conditions with low glucose (OM) or high glucose (OM, HG+). Fatty acid (FA), glucose, and glutamine (GLN) pathways were inhibited by Etomoxir (long chain fatty acid oxidation), UK5099 (glucose oxidation), and BPTES (glutamine oxidation inhibitor), respectively. Samples were prepared and differentiated as described in Section 2.8.1. FA dependency was tested by injections of 4μM Etomoxir followed by 2μM UK5099 along with 3μM BPTES. GLN dependency was tested by using 3μM BPTES followed by injections of 4μM Etomoxir along with 2μM UK5099. All the reagents were loaded in the ports as suggested by Agilent Technologies. A total of 15 measurements were taken to measure baseline conditions, followed by injection of target inhibitor and injection of the other two fuel inhibitors. Fuel dependency was calculated in percentage as followed:

Dependency fuel% = [(Baseline OCR–Target inhibitor OCR)/ (Baseline OCR–All inhibitors OCR) *100].

OCR rates were normalized by cell number and expressed as nmoles oxygen/min/1000 cells. One outlier was removed. All fuel dependency and targeted dependency pathways were calculated using Agilent Seahorse Wave Controller software.

### 2.9 Microscopy

Qualitative analysis of Alizarin Red S staining was performed using a 10X objective in Brightfield and optically imaged with Leica DMi1 by Leica Microsystems.

### 2.10 Statistical analysis

All experiments were performed in triplicates. All data are expressed as mean ± standard deviation. Comparisons of group means were performed by two-way analysis of variance (ANOVA) followed by Tukey posthoc to analyze the interaction of treatment and time or the interactions of various parameters of treatment/assay. Unpaired t-test was used when only two groups were involved, and Welch-correction was used when groups did not have equal variance. Effect size was determined using the Cohen’s D method. Each statistical analysis was performed using GraphPad Prism 6.0 (GraphPad Software Inc., La Jolla, CA, USA). A *p* < 0.05 was considered statistically significant.

## 3. Results

### 3.1. Inhibition of mineralization in bone cells undergoing glucose treatment

**Fig 1:**
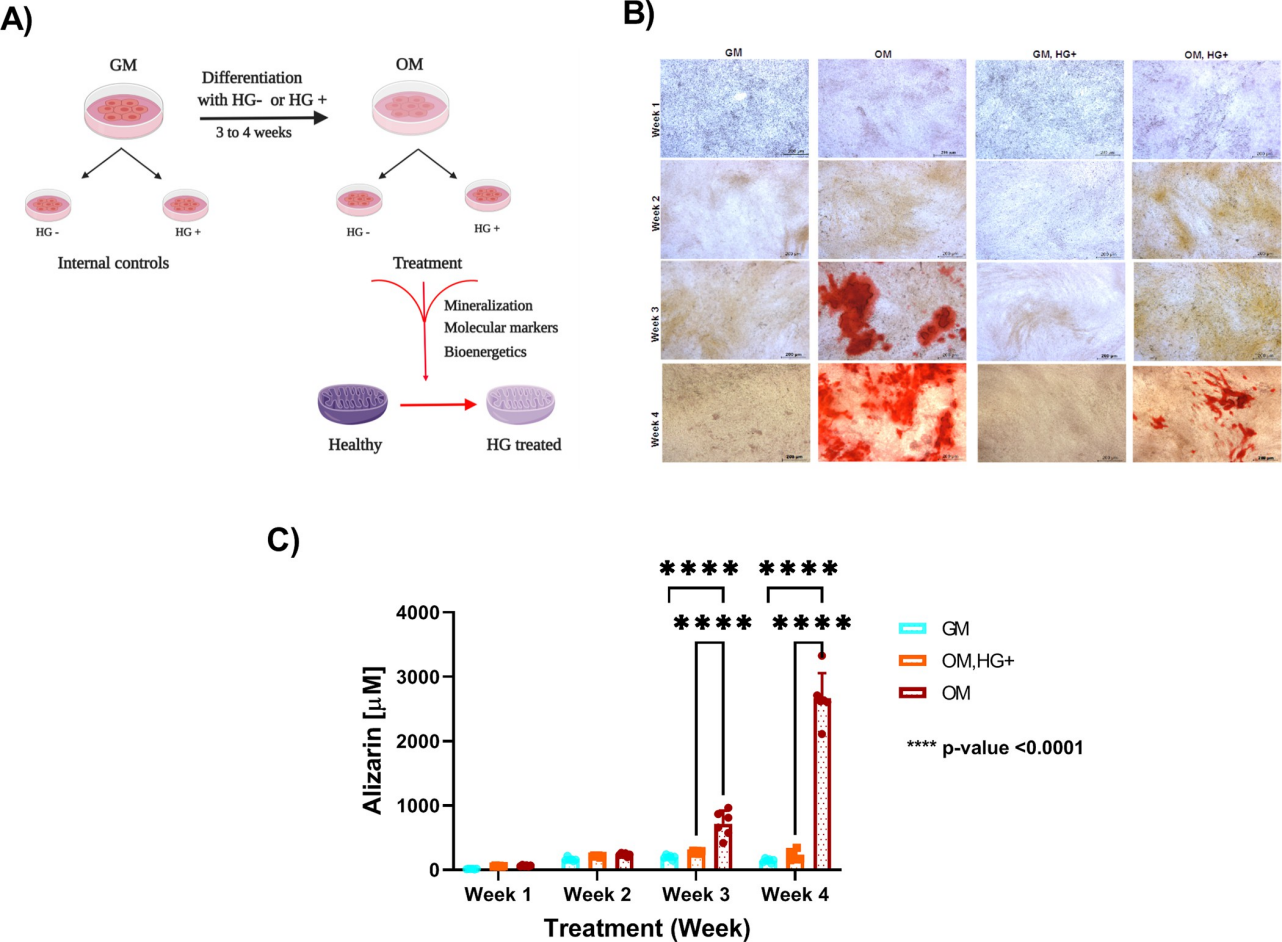
Alizarin S red staining of MC3T3 cells undergoing differentiation in the presence (OM, HG+) or absence of high glucose (OM). A. Schematic of Type 2 diabetes (T2D) protocol *in vitro*. B. Qualitative analysis of Alizarin S Red staining using one out of six biological replicates*. C. Quantitative analysis of Alizarin S red staining comparing MC3T3 nondifferentiated (GM) and differentiated (OM) cells. D. Quantitative analysis of Alizarin S red staining comparing differentiated MC3T3 cells under low glucose conditions (OM) and high glucose (OM, HG+) conditions. Error bars represent standard deviation (SD). Data were analyzed using Two-way ANOVA as described in methods. Abbreviations: GM = minimal growth media; OM = Osteogenic media; HG + = high glucose; HG- = low glucose. N = 6 per culture condition. One experiment was performed in triplicates per biological replicate at the end of each week for four weeks. *Created with BioRender.com.

Mineral formation in the extracellular matrix is a required phase for bone cell differentiation. Type 2 diabetes (T2D)- induced bone cells exhibit a reduction in the mineral phase formation [18, 19]. To further confirm that mineralization is reduced *in vitro*, calcium deposition was assessed by differentiation of pre-osteoblastic MC3T3 cells (OM media) and treated simultaneously in the presence of high (HG+) or low glucose (HG-) conditions for three or four weeks (Fig 1A). Nondifferentiated pre-osteoblast cells were cultured in GM media and treated under HG+ and HG-conditions as internal controls for three or four weeks in parallel to differentiation (Fig 1A). To quantify mineralization, Alizarin Red S staining was performed, a dye designed to stain calcium deposits in the matrix monolayer. Cells were stained at the end of each week for four weeks and alizarin concentration was recorded. Prior to quantification, wells were optically imaged. A small amount of calcium deposits was detected in two out of six biological replicates in differentiated cells under high glucose treatment (OM, HG+) only in the last week of treatment (Fig 1B), as a variation in biological replicates that had no statistical significance. Accumulation of calcium was seen only in osteoblast differentiated cells in low glucose environment at week 3 (OM) (Fig 1B). Differentiated cells (OM, HG-) displayed significant accumulation of calcium than nondifferentiated cells (GM, HG-) as expected (Fig 1C).Despite a slight detection of calcium content in two biological replicates, differentiated cells treated under HG conditions (OM, HG+) had a significant decrease of calcium content in comparison to differentiated cells (OM, HG-) treated under low glucose conditions (Fig 1C). The larger effect size suggests that the difference in calcium deposition with glucose treatment became more pronounced over time (Table 1). Nondifferentiated cells in the presence of low and high glucose were used as internal controls. As expected, nondifferentiated cells (GM) exhibited only background noise of alizarin concentration under high (GM, HG+) (S1A and S1B Fig) or low (GM, HG-) glucose conditions (S1A Fig). Together, these results suggest inhibition of calcium deposition at week 3 of glucose treatment and, consequently inhibition in osteoblast differentiation.

**Table 1:** The magnitude of effect size increased with time as MC3T3_E1 cells were treated with high glucose during differentiation.

Effect Size				
**Alizarin [μM]**	Week 1	Week 2	Week 3	Week 4
**OM, HG+ vs OM**	-0.92675	-1.6976	-3.055	-8.73

#### 3.1.1. High glucose treatment promotes biochemical defects in osteoblasts

**Fig 2:**
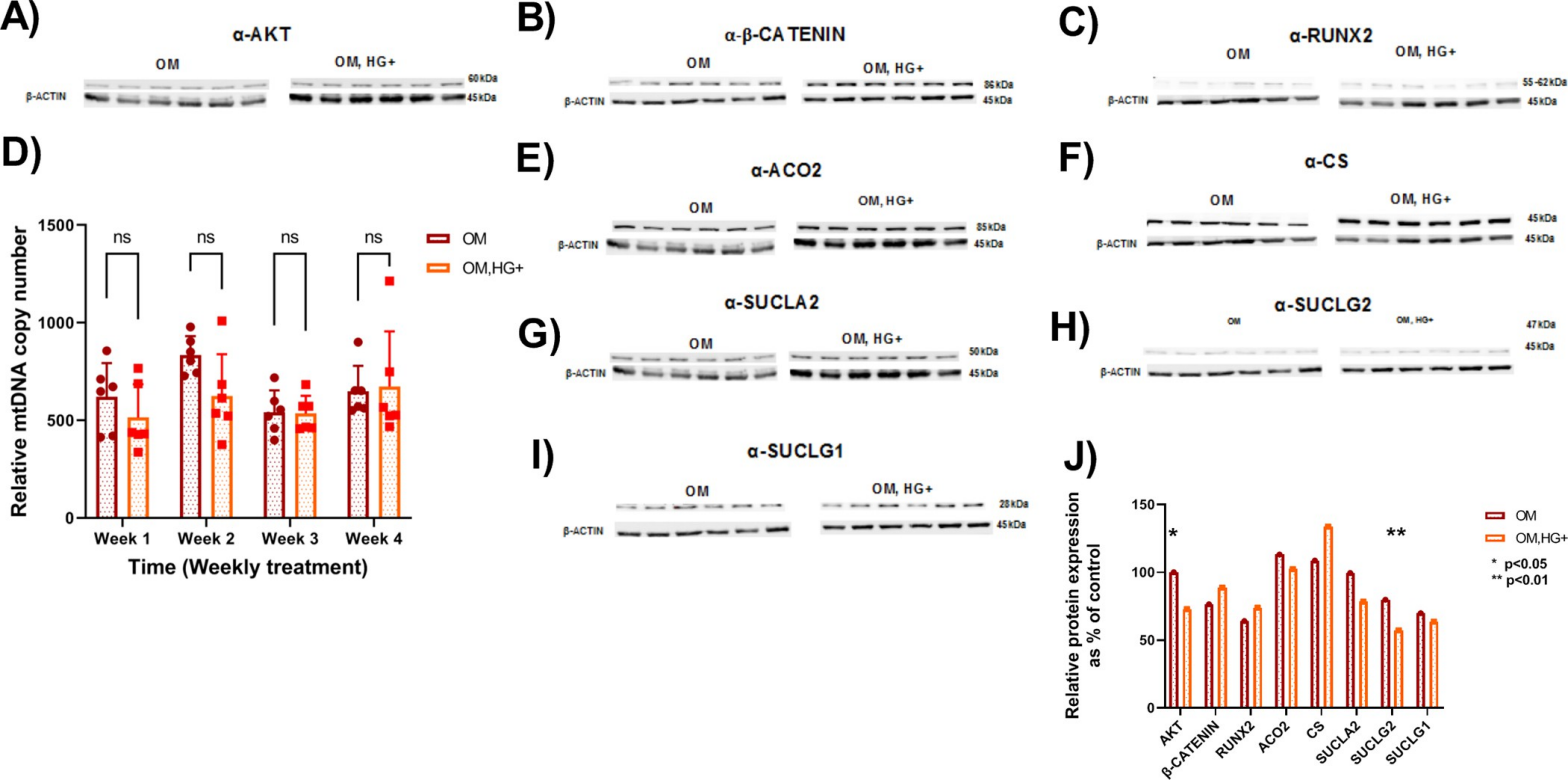
Molecular characterizations of differentiated MC3T3-E1 cells demonstrate altered markers in the presence of high glucose. A. Immunoblot analyses of AKT. B. Immunoblot analyses of β-Catenin. C. Immunoblot analyses of RUNX2. D. mtDNA content was reduced in HG-treated bone cells. E. Immunoblot analyses of ACO2. F. Immunoblot analyses of CS. G. Immunoblot analyses of SUCLA2. H. Immunoblot analyses of SUCLG2. I. Immunoblot analyses of SUCLG1. J. Relative protein expression as percentage of control. All protein lysates for western blot experiments were treated in the presence of low or high glucose for three weeks (week3). Densitometry was measured using ImageJ software and normalized with loading control. Student t-tests were used for data analysis. Asterisk indicates p-value <0.05. Abbreviations: OM = differentiated cells under low glucose conditions; OM, HG+ = differentiated cells under high glucose conditions. Data represent N = 6 independent experiment per culture condition using one technical replicate.

Given that the mineralization is inhibited in differentiated cells under high glucose conditions, it was hypothesized that proteins involved in osteoblast differentiation would be altered at the protein level as well. Osteoblast differentiation involves several proteins from distinct signaling pathways (PI3K/Akt and WTN/β-Catenin) that regulate RUNX2, the master regulator of osteoblast differentiation [32]. In addition to bone differentiation, PI3K/Akt pathway regulates various cellular processes including glucose homeostasis [33]. Previous work in both rat diabetic pancreatic β cells and mouse diabetic osteoblast cells showed a decrease in the ratio of pAKT/total AKT expression [18, 34]. To determine biochemical defects in HG-treated osteoblasts, the expression of RUNX2-mediated osteoblast differentiation regulators was examined in differentiated cells in the presence of low or high glucose treatment at later stages of differentiation. The protein expression of total AKT was significantly reduced (p = 0.04) in differentiated cells under HG conditions during week 3 of treatment (Fig 2A and 2J). Conversely, total AKT expression was significantly elevated (p = 0.03) in HG-treated bone cells during week 4 of treatment (S2A and S2J Fig). Although changes in β-CATENIN (Fig 2B and 2J) and RUNX2 (Fig 2C and 2J) were observed, these trends were not significant suggesting that other osteoblast differentiation markers via RUNX2 might be affected under HG conditions.

#### 3.1.2. High glucose treatment leads to mitochondrial biochemical defects in osteoblasts

mtDNA content was quantified to detect depletion, which is considered a hallmark for many disease states. At the molecular level, accumulation of mutations in mtDNA leads to mtDNA depletion in T2D [35, 36]. To estimate mtDNA in HG-treated bone cells, we quantified mtDNA copy number in differentiated cells for four weeks of low and high glucose exposure using qPCR. At each time point, mtDNA copy number in HG-treated osteoblast cells remained similar to controls and although values trended lower, the decrease was not significant (Fig 2D and S2D Fig). As altered total AKT, a regulator of RUNX2 biochemical marker, expression was detected in HG-treated osteoblasts, the potential link between osteoblast and mitochondrial function in the context of T2D *in vitro* was explored. T2D is a metabolic disorder known to display bioenergetics defects with a decrease in expression of mitochondrial markers [16, 37] in various tissues [38] promoting oxidative stress and mitochondrial dysfunction [39]. Because previous work examined mitochondrial genes associated with OXPHOS and mitochondrial fission and fusion, the focus of this study was to investigate the biochemical role of key enzymes of the Tricarboxylic acid (TCA) cycle, the central metabolic pathway of cells, in HG-treated osteoblasts. The expression of citrate synthase (CS), mitochondrial Aconitase2 (ACO2), and Succinyl-CoA synthetase (SCS), all TCA cycle enzymes, were explored. CS is responsible for the second step of the TCA cycle that catalyzes Oxaloacetate (OAA) and Acetyl-CoA to citrate and, therefore, is considered the key biochemical marker for mitochondrial matrix content (mitochondrial mass). In HG-treated bone cells, both the increase of CS expression at week 3 (Fig 2F and 2J) and decrease at week 4 of treatment (S2F and S2J Fig) were not significant. Next, immunoblots were performed to detect ACO2 enzyme expression. ACO2 is responsible for the conversion of citrate into α-ketoglutarate coupled with NADP reduction and used as an indirect measure of reactive oxygen species (ROS) accumulation. In HG-treated bone cells, ACO2 expression had no significant changes at both week 3 (Fig 2E and 2J) and week 4 (S2E and S2J Fig) of treatment. After that, Succinyl-CoA synthetase (SCS) was investigated. SCS is an enzymatic complex comprised of an α-subunit encoded by SUCLG1 gene and the β-specific dNDP subunit (encoded by either SUCLA2 or SUCLG2 in tissue specific pattern of expression) [30]. SCS is responsible for the only step of the TCA cycle that produces ATP. In HG-glucose bone cells, SUCLA2 expression was nonsignificant reduced at week 3 (Fig 2G and 2J) followed by a significant increase (p = 0.016) at week 4 (S2G and S2J Fig). SUCLG2 expression showed a similar pattern to SUCLA2 expression with a significant reduction (p = 0.0069) (Fig 2H and 2J) and elevation (p = 0.0016) (S2H and S2J Fig) in weeks 3 and 4 of treatment, respectively. Finally, SUCLG1 expression remained similar at both weeks 3 (Fig 2I and 2J) and 4 (S2I and S2J Fig).

Taken together, these findings suggest changes in expression of total AKT and mitochondrial SCS expression, specifically the β-specific dNDP subunits, along with a trend towards mtDNA depletion confirming that both biochemicals markers are affected due to exposure to high glucose treatment at later stages of differentiation. For this reason, the function of key mitochondrial enzymes was investigated.

### 3.2. Osteoblast cells exhibit mitochondrial perturbation under HG conditions

**Fig 3:**
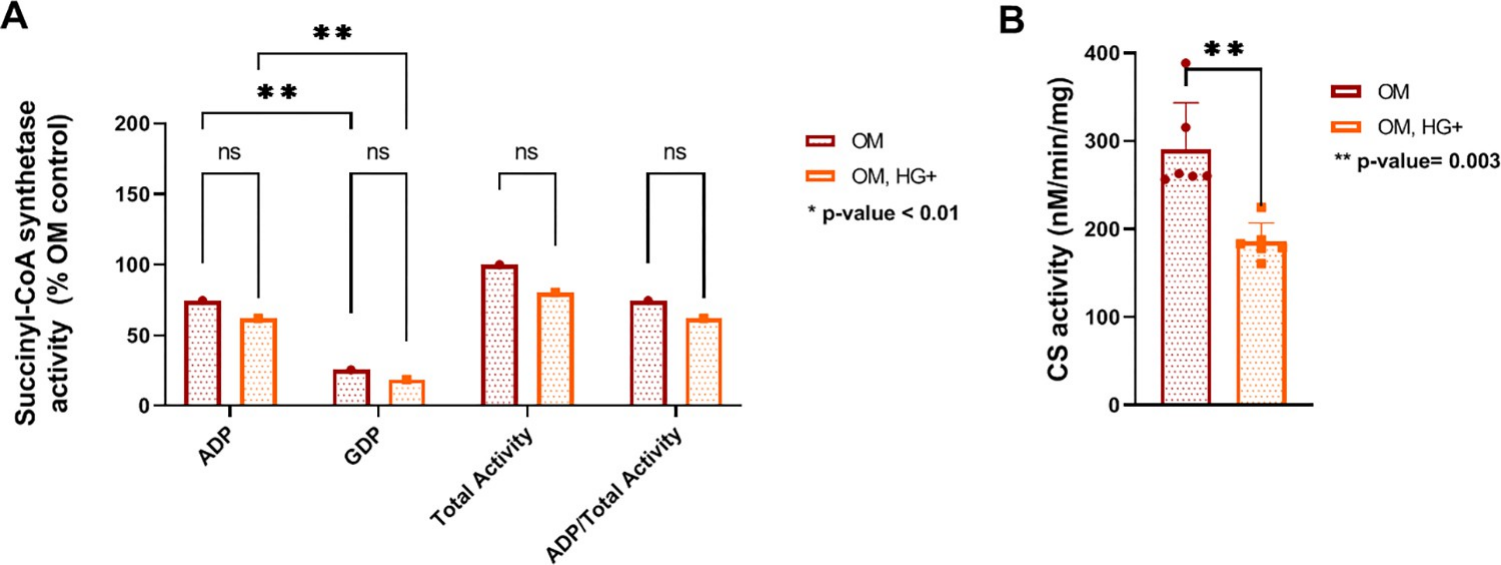
Key enzymes of the TCA cycle have reduced activity in HG-treated bone cells. A. Succinyl-CoA synthetase activity was calculated by measuring each substrate (ADP or GDP) and was expressed as percent total activity for each condition independently. Rates for Succinyl- CoA synthetase activity were calculated by measuring each substrate (ADP and GDP) and correcting for absence of either ADP or GDP. After correction, ADP and GDP activities of both OM and OM, HG+ cells as percent of total activity in OM cells. B. Citrate synthase activity of differentiated cells was calculated in the absence and presence of HG. CS activity data represent two independent experiments. All experiments were performed in triplicates. CS activity was analyzed by Student T-test paired-wise comparison. SCS activity was analyzed by Two-way ANOVA. CS activity data correspond to two technical replicates of N = 6 independent experiments in triplicates.

Due to the significant changes in expression of key TCA enzymes, it was hypothesized that the function of these enzymes was altered under HG conditions. First, ADP-specific and GDP-specific SCS activities were measured in differentiated cells in high (OM, HG+) or low (OM) glucose. ADP-specific activity was significantly higher ithan GDP- specific SCS activity in both controls (OM) and HG- treated cells (Fig 3A) suggesting that bone cells have a preference for ADP-specific substrate. In SCS deficiency, reduction of ADP- specific activity was compensated for by elevation of GDP- specific activity, which maintained total SCS activity in the disease state versus controls [31]. In this study, however, there was no compensation of GDP-specific subunit. HG-treated osteoblast cells displayed a nonsignificant reduced activity of both dNDP- specific SCS subunits, and consequently a trend toward reduction of total SCS activity (Fig 3A). Next, CS activity was measured. CS is a marker for mitochondrial matrix content and altered CS activity has been reported in T2D [40]. In HG-treated bone cells, the reduction of citrate synthase activity was significant in comparison to controls (Fig 3B). Together, these data demonstrate that osteoblasts prefer ADP-specific subunit as substrate, and intact mitochondrial matrix content is reduced as a result of a significant decrease in CS activity in bone cells and as a consequence of the negative effects of high glucose treatment.

### 3.3. HG-treated bone cells utilizes both mitochondrial respiration and glycolysis for cellular energy requirements

**Fig 4:**
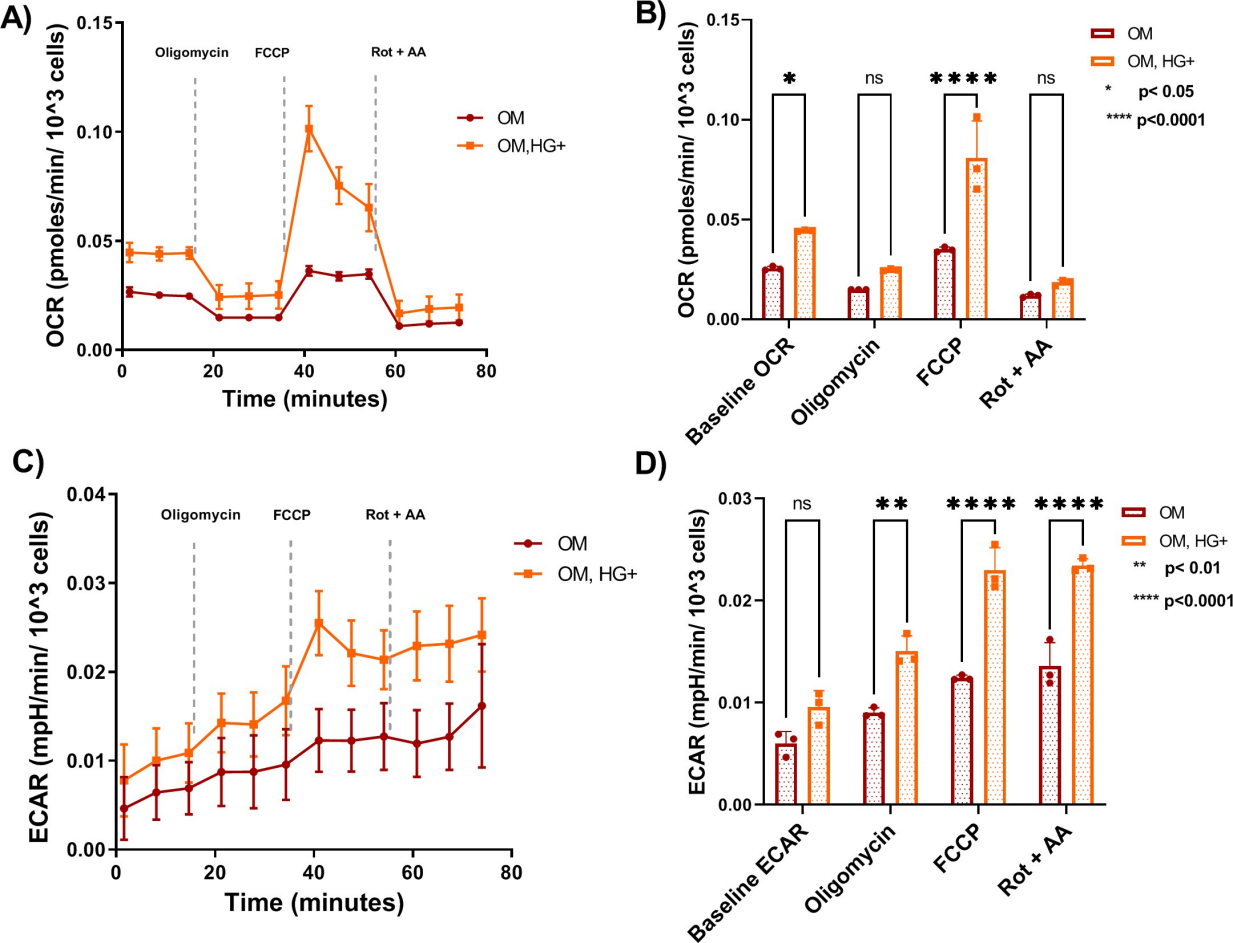
HG-induced osteoblast cells utilize both mitochondrial respiration and Glycolysis more efficiently than healthy cells. A. Oxygen consumption rate is indicative of mitochondrial respiration in healthy and HG-treated cells. B. Four channels of sequential injections exhibited an increase in OCR levels in HG-treated bone cells. C. Extracellular acidification rate is indicative of glycolysis in healthy and HG-treated cells. D. Four channels of sequential injections exhibited an increased in ECAR levels in HG-treated bone cells. Abbreviations: OCR = Oxygen Consumption Rate; ECAR = Extracellular Acidification rate. Error bars indicate SD (standard deviation). Data were analyzed using Two-way ANOVA. A total of N = 3 independent experiments per culture condition were performed. All experiments were performed in triplicates.

**Fig 5:**
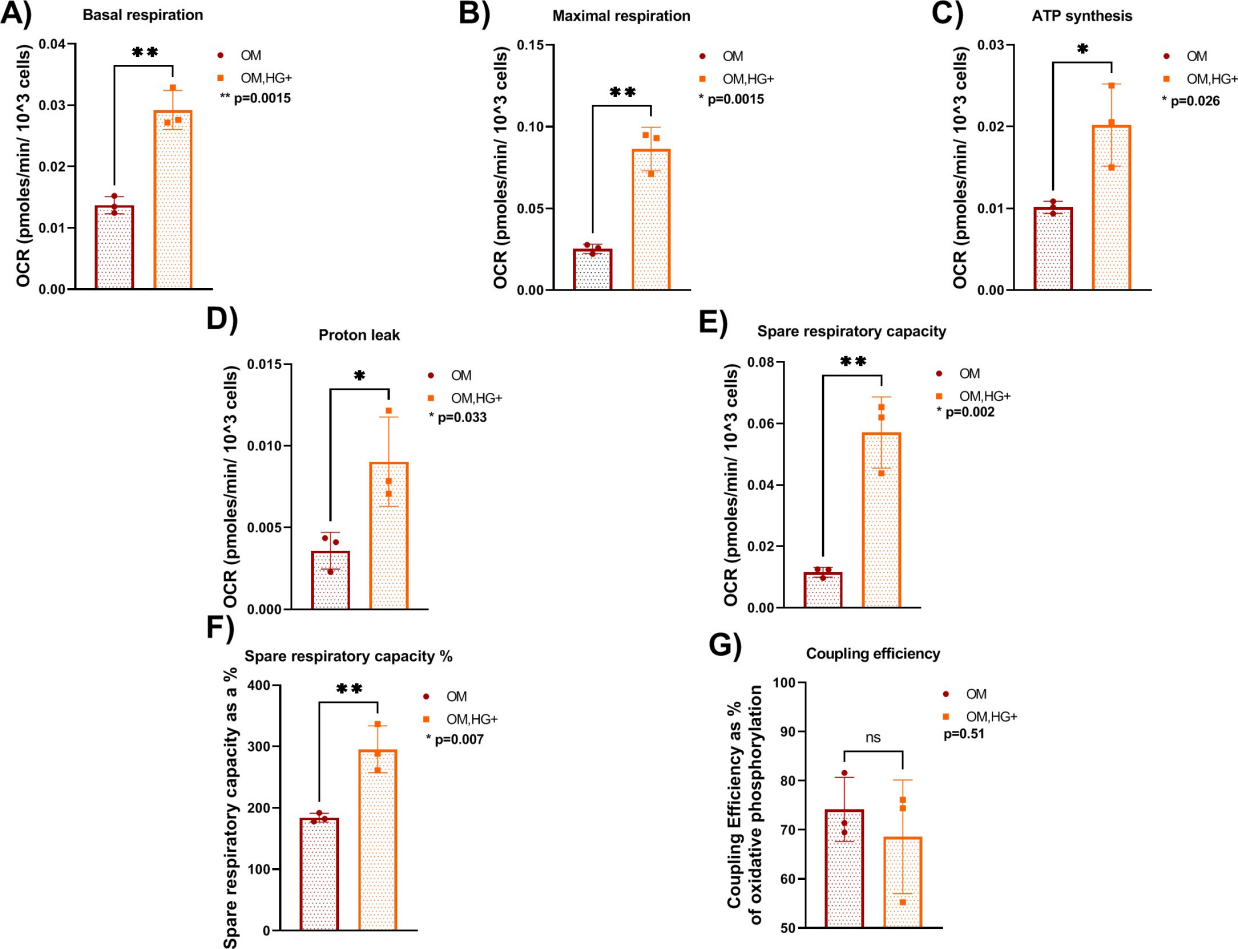
Fundamental parameters of mitochondrial respiration are elevated in HG-treated bone cells. A. Basal respiration. B. Maximal respiration. C. ATP synthesis. D. Proton leak. E. Spare respiratory capacity. F. Spare respiratory capacity %. G. Coupling efficiency. Error bars indicate SD (standard deviation). Data were analyzed using Student T-test. A total of N = 3 independent experiments per culture condition were performed. All experiments were performed in triplicates.

Energy metabolism shifts have been reported in the context of diabetes [23, 27, 41]. Mitochondrial respiration is the metabolic process that requires oxygen consumption to generate energy at the cellular level. Differentiation of osteoblasts requires oxidative phosphorylation as well as glycolysis, with the latter considered necessary for osteoblast differentiation [42]. In past studies, distinct cell lines were examined for energetic demands under diabetic conditions. Human diabetic peripheral blood [43] as well as mouse pancreatic β-cells [44] exhibited altered mitochondrial respiration. To determine bioenergetics demands in T2D-induced osteoblast cells, mitochondrial respiration was assessed using the Seahorse mito stress assay. Interestingly, the results demonstrated that HG-treated osteoblasts displayed a 1.6 to 3-fold increase of OCR levels in comparison to healthy cells (Fig 4A) with significant increased baseline OCR (Fig 4B) suggesting a preference for OXPHOS to meet energy demands. Both baseline respiration and FCCP-stimulated maximal respiration were significantly increased in HG-treated cells in comparison to controls (Fig 4B). Furthermore, the mito stress test provides information on Extracellular acidification rate (ECAR) levels, a measure of glycolysis utilization. Both healthy and HG-treated osteoblasts utilized glycolysis to meet their energy demands (Fig 4C). Most ECAR channels significantly increased in HG-treated osteoblast cells in comparison to controls (Fig 4D). To further examine the OCR results, we examined all fundamental parameters of mitochondrial respiration. Basal respiration is the minimal rate of oxygen consumption needed to maintain the cell’s function. The OCR levels of basal respiration (Fig 5A) and proton leak (Fig 5D) significantly increased exhibiting double OCR levels in treated cells compared to controls. Interestingly, the significant increase of proton leak suggest an incomplete coupling of oxygen substrate to generate ATP and potential mitochondrial damage. Maximal respiration is the cell’s ability to reach maximal oxygen consumption after stressed by FCCP, to meet energy demands at rapid oxidation rate. Maximal respiration (Fig 5B), as well as spare respiratory capacity (Fig 5E) increased significantly with OCR levels reaching three to five times higher levels, respectively, in differentiated osteoblastic cells under HG conditions in comparison to controls. A 2-fold increase of ATP synthesis (Fig 5C) and 1.5-fold significant increase of spare respiratory capacity % were detected in HG-treated osteoblasts compared to controls (Fig 5F). The significant elevation of spare respiratory capacity and spare respiratory capacity %, and ATP synthesis confirmed that the ability of HG-treated bone cells to reach energy demands is via OXPHOS. The increase in coupling efficiency in bone cells under HG conditions was not significant (Fig 5G) in HG treatment in comparison to controls. Overall, these results suggest that HG-treated osteoblasts cells utilize both OXPHOS and glycolysis with preference for cellular respiration indicating significant shifts in Bioenergetics from normal conditions. For this reason, further assessment of metabolic activity was pursued.

### 3.4. Osteoblast cells exhibit an increased oxygen consumption, in part, supported by FA oxidation under HG-induced conditions

**Fig 6:**
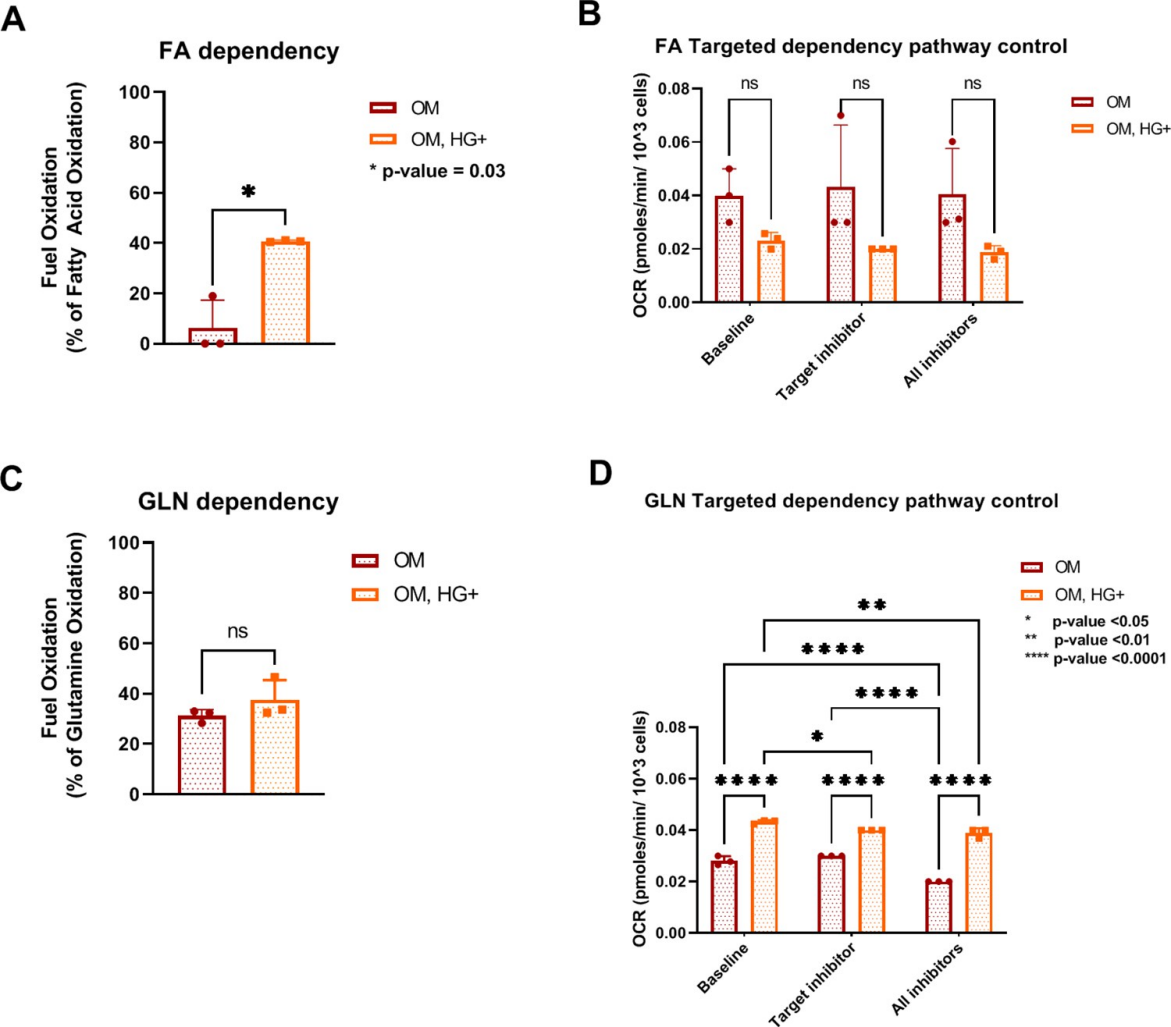
Osteoblast cells display FA dependency for mitochondrial oxidation under HG-induced conditions. A. FA oxidation dependency. B. FA targeted pathway dependency control. C. GLN oxidation dependency. D. GLN targeted pathway dependency control. Error bars indicate SD (standard deviation). Data were analyzed using Two-way ANOVA. N = 3 independent experiments were performed per condition. All experiments were performed in triplicates. Abbreviations: FA = fatty acid; GLN = glutamine.

After confirming HG-treated cells’ preference for mitochondrial respiration, the fuel needs of differentiated cells treated under low or high glucose conditions were assessed. Previous work demonstrated that glucose oxidation decreased OCR and, in contrast, increased ECAR suggesting that differentiated osteoblasts prefer glycolysis over OXPHOS to meet bioenergetic requirements [20]. As demonstrated here, key parameters of glycolysis were significantly increased (Fig 4D). However, ECAR levels only increased approximately 1.5-fold after FCCP injection (Fig 4B). Next, we sought to explore other fuels as potential sources of oxidation in HG-treated bone cells and controls. To investigate fuel oxidation, dependency and capacity of cells can be measured. As a shift in metabolic needs was detected, dependency was investigated. Dependency of mitochondrial oxidation measures the cells’ reliance on a fuel to maintain the minimal (baseline) requirements to achieve the energy demands. As described in methods, dependency is measured after injection of specific pathway inhibitors that can act by decreasing (affecting the pathway utilization) or increasing (no effect in using the pathway for energy production) the OCR levels corresponding to each step of the specific pathway (s) inhibited known as targeted dependency pathway. First, we assessed Fatty acid (FA) oxidation dependency. FA is one of the main fuels for the body yielding more energy than glucose utilization. FA β-oxidation generates by-products including Acetyl-CoA and NADH via reducing reactions for the TCA cycle and Complex I of OXPHOS, respectively [23, 45]. Our results detected a significant increase in FA dependency under HG conditions (Fig 6A) with a decrease of targeted dependency pathway presented in HG-glucose bone cells (Fig 6B) in comparison to controls. The baseline OCRs were rather low in HG-treated osteoblasts potentially due to disruption of matrix monolayer during the assays. Additional work will be required to confirm our initial findings that unlike healthy osteoblasts, HG-treated osteoblasts rely on FA β-oxidation during differentiation.

Next, additional fuel oxidation was performed by examining Glutamine (GLN) oxidation. Glutamine is an important amino acid to yield energy to the TCA cycle [46] and reduce oxidative stress [47]. In HG-treated osteoblast cells, there was a nonsignificant increase in GLN dependency suggesting HG-treated osteoblasts do not depend on glutamine for fuel oxidation (Fig 6C), but rather have the ability to utilize GLN as fuel source (metabolic capacity). Additionally, the key parameters tested for the targeted dependency pathway were significantly increased in HG-treated osteoblasts in comparison to controls suggesting that HG-treated osteoblast cells require more than one fuel source for mitochondrial oxidation compared to controls (Fig 6D).Together, these data suggest that osteoblast cells rely in FA and other sources (fuels or amino acids) to maintain the baseline energetic needs under hyperglycemic conditions.

## 4. Discussion

In this study, we confirmed that T2D affects mineralization, alters expression of bone and mitochondrial proteins, decreases mitochondrial matrix content, and shifts metabolic needs as an indication of mitochondrial dysfunction in differentiated osteoblast cells. Calcium deposition was inhibited in cells treated with high glucose (HG). This inhibition was detected in previous studies [18, 19, 48]. Few calcium deposits might be associated with the decrease in calcium uptake previously detected in HG-treated osteoblasts [49]. Overall, the inhibition of mineral deposition might be related to structural and functional impairment of osteoblast cells due to hyperglycemia. In recent studies, it was revealed that citrate binds to the hydroxyapatite crystal structure [50, 51] and mediates mineral deposition during osteoblast differentiation [52]. It is possible that citrate levels are accumulating in the cytosol of HG-treated cells. In diabetic rats and humans, there was an increase of plasma citrate suggesting that accumulation of citrate is indicative of altered TCA cycle intermediates [53]. Moreover, high levels of citrate in the cytosol inhibit enzymes involved in glycolysis [54] and reduce mineral deposition [52]. Further studies are warranted to determine additional substrate requirements for mineralization and if they are affected in T2D of bone cells.

The inhibition of calcium deposition induced by HG treatment at later stages of differentiation affect molecules involved in differentiation and/or metabolism. Altered expression of β-CATENIN and RUNX2 may be more critical in other diabetic tissues [55–57]. Diabetic skeletal muscle contributes to decrease in bone quality. In skeletal muscle of *Akt*-deficient mice, the lack of AKT expression suggested that AKT2 isoform was responsible for glucose uptake impairment, a feature of T2D, specifically in the skeletal muscle tissue [58]. In previous work, AKT expression exhibited similar altered expression with significant changes in total AKT expression [59]. Dong and colleagues [18] detected a significant decrease in expression of phosphorylated AKT (pAKT) to total AKT ratio. The phosphorylated form of AKT is responsible for regulation of glucose uptake via glycolysis [60]. In addition, AKT modulates ATP citrate lyase (ACLY) to generate Acetyl-CoA used to produce FA in mouse adipocytes [61]. The role of AKT in glucose uptake and FA metabolism in bone cells warrants further investigation. Additional studies are warranted to investigate AKT phosphorylated states and distinct isoforms as well as measure phospho AKT/ total AKT ratio as well as examine the role of upstream molecules in the pathway that modulate AKT function under HG conditions.

Altered AKT expression and inhibited calcium deposition in HG-treated bone cells suggested altered formation and function of the mineral phase with potential changes at the DNA level. Janssen and colleagues [62] identified a mtDNA mutation associated with mitochondrial dysfunction and low heteroplasmy in pancreatic islet cells [63] derived from diabetic patients. In diabetic patients, aging was also associated with mtDNA depletion [64]. In HG-treated osteoblast cells, a trend towards mtDNA depletion was seen, but not confirmed statistically demonstrating that HG treatment did not promote mtDNA depletion. In past work, mtDNA content was decreased only in diabetic islet cells from old mice [13]. As T2D is more pronounced with aging, future studies might consider quantifying mtDNA in cells from higher passage number, longer treatment, and/or cells derived from older diabetic animals to detect a reduction in mtDNA copy number *in vitro*.

In diabetes, mitochondria displayed a downregulation of OXPHOS genes [16] as well as a reduction in OXPHOS proteins in skeletal muscle [65]. Here, the focus was to examine the expression of enzymes involved in the TCA cycle. In HG-treated osteoblasts, expression of CS was not significantly reduced. Additionally, the expression of both β-specific dNDP subunits (SUCLA2 and SUCLG2) were significantly altered. In pancreatic pre-diabetic and diabetic cells, the expression of all enzymes of the TCA cycle were reduced including all subunits of SCS [44]. It is possible that another pathway might be supporting the TCA cycle to compensate for changes in protein expression throughout cell differentiation under HG conditions. It will be important to investigate intermediates/metabolites of the pathway to determine if other steps are affected.

To confirm mitochondrial perturbations, the enzymatic functions of both CS and SCS were assessed in this study. To our knowledge, this is the first time SCS activity is investigated in osteoblasts. SUCLA2 is highly expressed in catabolic tissues such as brain, heart, and skeletal muscle [30]. Interestingly, osteoblast cells have a substrate preference for ADP-specific subunit instead of GDP- specific subunit. In hyperglycemic conditions, both ADP-specific and GDP-specific, as well as total SCS activity, were reduced but failed to reach significance with no dNDP-specific subunit compensation, as seen in a previous study [31]. Conversely, CS activity was significantly compromised in HG-treated bone cells. The reduction of CS activity was reported in both diabetic skeletal cells [40] as well as human diabetic muscle [14, 66] as well. Overall, our study demonstrated that mitochondrial matrix content was reduced as CS activity is considered a more reliable marker for mitochondrial content than the mtDNA copy number [67]. In future studies, protein content and function will be assessed in isolated mitochondria to further examine mitochondrial alterations.

Mitochondria regulate bioenergetics needs during differentiation [68]. Changes in metabolic preference have been reported in human adipocytes [69], cardiac cells [70], and osteogenic cells [71]. In murine stem cells, the differentiation process to adipocytes or osteocytes requires an increase in oxygen consumption [72] indicating metabolic shifts as well. Glycolysis is the main metabolic pathway for glucose utilization in healthy differentiated osteoblasts [20, 21]. In diabetic patients, skeletal muscle cells displayed a reduction in mitochondrial respiration [73]. Mitochondrial respiration is impaired in murine glial cells [74] as well as in pancreatic cells [44] treated under hyperglycemic conditions. The increase of both OCR and ECAR levels seen in HG-treated bone cells (week 3 of treatment) were detected in early stages (days 7 and 14) of differentiation [20, 42] and increase of OCR levels were detected in non-differentiated cells [20] supporting our findings that hyperglycemia leads to inhibition of osteoblast differentiation that affects not only mineralization, but promotes metabolic shifts. Most fundamental parameters of mitochondrial respiration displayed a significant increase in oxygen consumption. The elevation of mitochondrial respiration parameters suggest that HG-treated bone cells met energy demands for ATP production via oxidative phosphorylation similarly to what has been reported during early stages of osteoblast differentiation [20, 42] and potentially supported by another nutrient. One argument could be made that supplemented glucose present in XF assay media was responsible for the bioenergetic changes detected here, which would suggest assessment of bioenergetic needs was affected. However, past studies have demonstrated that osteoblasts by the end of week 3 of differentiation prefer glycolysis in the presence [42] or removal [20] of supplemented glucose. In addition, our group used less than half of the glucose concentration used by Guntur and colleagues [42] and one third concentration used for HG treatment in this study.

In T2D, adipocyte cells displayed an increase in mitochondrial respiration and fundamental parameters [75] in a similar manner identified in HG-treated osteoblasts in the current study. However, FA oxidation was not responsible for changes in mitochondrial respiration of diabetic adipocyte cells. The possibility that bioenergetic shifts are dependent on multiple fuel utilization in HG-treated bone cells is an intriguing hypothesis that would be interesting to explore further. Glucose utilization and FA oxidation share Acetyl-CoA production as a common step in their metabolic processes. FA utilization generates more energy than glucose production. FA oxidation is one of the main substrates to generate ATP in heart and skeletal muscles [24, 76] by producing Acetyl-CoA to fuel the TCA cycle, reducing FADH_2_ and NADH into the Electron transport chain (ETC) of OXPHOS [23]. In bone cells, palmitate (FA) oxidation produced more ATP than oxidation of either glucose or lactate [77]. With that in mind, dependency of FA oxidation was investigated in osteoblasts to determine if HG-treated bone cells depend on FA as the main fuel to meet metabolic demands. Dependency of HG-treated bone cells on FA as fuel was significant. However, it will be important to examine intermediates for FA oxidation in HG-treated bone cells as well as assess dependency along with capacity to utilize FA for oxidation concurrently to measure HG-treated osteoblasts FA flexibility, which will determine if osteoblasts under hyperglycemic conditions have the ability to compensate for the inhibited pathway by utilizing other pathways for mitochondrial respiration.

Although HG-treated osteoblasts rely on FA utilization to maintain baseline energy needs, other fuels might contribute to increase of OCR levels. Therefore, glutamine (GLN) dependency was examined in HG-treated bone cells. Glutamine is an amino acid that contributes to the TCA cycle and FA synthesis by generating α-ketoglutarate, an intermediate of the TCA cycle that is also used to generate citrate in the cytosol for FA synthesis [47]. In bone cells derived from mice deficient in glutaminase (GLS1), glutamine is essential for osteoblast proliferation and survival along with matrix formation, differentiation, and consequently mineralization [78]. Our study revealed an increase in GLN dependency in HG-treated bone cells, albeit not significant. All parameters measured in GLN targeted pathway were significantly increased in HG-treated bone cells in comparison to controls suggesting utilization of other fuels to maintain minimal metabolic demands for cell function. Furthermore, OCR levels were significantly increased in HG-treated bone cells in comparison to controls during the glutamine fuel test. These data suggest that HG-treated bone cells do not depend on glutamine but might display capacity and flexibility to utilize GLN and might be dependent on other substrates for mitochondrial oxidation. Further work is warranted to determine both capacity and flexibility of Glutamine and FA oxidation in HG-treated osteoblasts as well as to identify dependency of other substrates for mitochondrial oxidation.

One limitation of the HG-treatment in osteoblasts *in vitro* reduce mineral content in contrast to what has been reported in humans. Another limitation of our study is that lack a control with carbohydrates to maintain osmolarity may affect results due to high osmolarity. Although in a previous work with mannitol control by Balint and colleagues (2001) the calcium deposition was inhibited under HG conditions. One more limitation here is that expression and function of mitochondrial TCA enzymes was assessed in whole lysates, which could have diluted out the results, and masked significance of altered SCS enzymatic activity important for mitochondrial function. Lastly, there is a limitation with unforeseen issues related to matrix monolayer detachment during FA oxidation studies, which contributed to reduced baseline OCR levels in HG-treated osteoblasts.

Additional studies are required to determine how high glucose directly acts in bone cell differentiation and metabolic flexibility. One possibility is that two pathways act in parallel by affecting TCA intermediates that connects Glucose metabolism to FA oxidation [23] or via impairment of PI3K/Akt signaling in triggering AKT function to control glucose and FA synthesis [79]. Therefore, it will be important to investigate if other molecules such as citrate are involved and accumulating at high levels in the cytosol. High levels of citrate impair differentiation [52], inhibit intermediate steps of glucose breakdown and inhibit upstream modulators of AKT signaling pathway in cancer cells [54]. AKT, in turn, is known to activate glucose uptake and utilization via glycolysis as well as activating ACLY to catalyze citrate and generate acetyl-CoA for FA production [79]. Another possibility is that multiple pathways and amino acids might be important for metabolic reliance to maintain baseline needs in HG-treated osteoblast cells. Determining the molecule(s) of interest will be crucial to elucidate the pathogenic mechanism for T2D in bone cells and potentially in other metabolic bone disorders exhibiting mitochondrial dysfunction.

**Fig 7:**
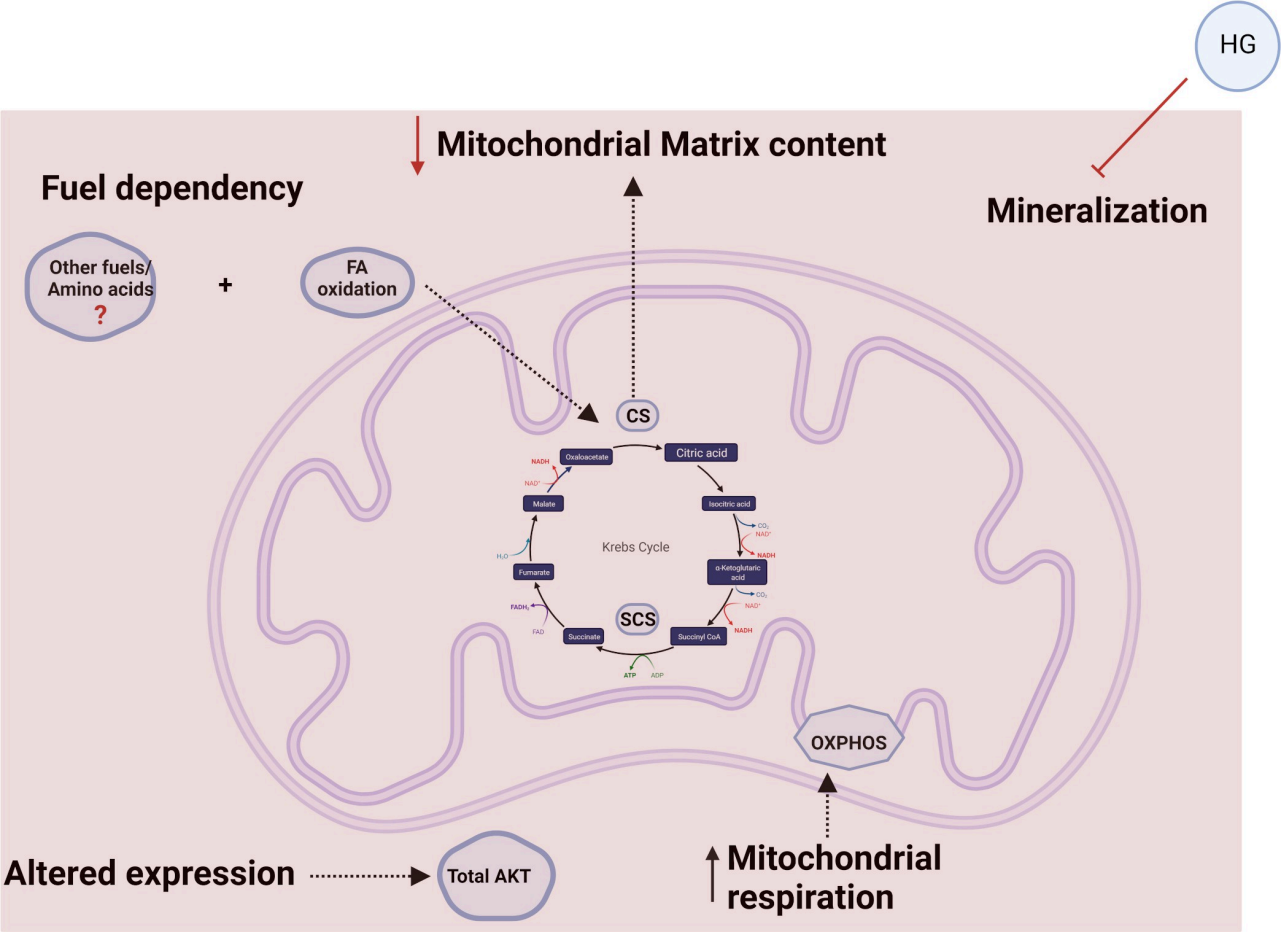
HG-treated osteoblast cells exhibit inhibited mineralization and changes in bioenergetic needs as a result of hyperglycemia inhibiting osteoblast differentiation. Accumulation of calcium was inhibited due to high glucose treatment. Total AKT expression was reduced as well. High glucose treatment promoted mitochondrial perturbations by altering expression of SCS subunits in the TCA cycle and reduction in mitochondrial matrix content via decrease of CS activity. Differentiated osteoblasts under hyperglycemic conditions utilize both mitochondrial respiration and glycolysis similarly to what is seen in early stages of differentiation suggesting that excess of glucose promotes shifts in metabolic needs with preference for mitochondrial respiration. HG-treated osteoblast cells rely on fatty acid to maintain baseline energy demands, but it seems other pathways are required in HG conditions. Molecules and metabolic pathways altered under high glucose conditions are depicted in light blue. *Figure was created by BioRender.com.

In conclusion, we developed an *in vitro* system to examine T2D in bone cells which exhibited a decrease in calcium deposition, a reduction in the expression of AKT, SUCLA2, and SUCLG2 along with a decrease in mitochondrial content (reduction of CS activity) and a shift in metabolic needs from only using Glycolysis to using both glycolysis and OXPHOS with minimal energy requirements supported by FA pathway and other pathways yet to be determined (Fig 7). To our knowledge, this is the first study to investigate mitochondrial respiration and TCA enzymes as well as Fuel oxidation in osteoblast cells in an environment mimicking T2D. Taken together, our findings suggest that HG-treated osteoblasts display inhibited mineral phase with mitochondrial perturbations as well as altered metabolic needs similar to the bioenergetics of undifferentiated and early differentiated bone cells. Future work will focus on PI3K/Akt signaling pathway as well as common intermediates of metabolic pathways to help elucidate the molecular mechanisms of T2D in bone *in vitro*.

## Supporting information

S1 Fig Alizarin S red staining of undifferentiated MC3T3 cells in the presence of HG. A. Quantitative analysis of Alizarin S red staining comparing undifferentiated MC3T3-E1 cells in the absence (GM)or presence of HG (GM, HG+). B. Quantitative analysis of Alizarin S red staining comparing undifferentiated (GM) and differentiated (OM) MC3T3 –E1 cells in the presence of HG (HG+). Assay were performed in triplicates at the end of each week for four weeks. Error bars represent standard deviation (SD). Abbreviations: GM = minimal growth media; OM = Osteogenic media; HG = high glucose, 30.5 mM final concentration.
(TIF)

S2 Fig Molecular characterization of MC3T3-E1 cells demonstrate altered markers in the presence of high glucose. A. Immunoblot analyses of AKT. B. Immunoblot analyses of β-CATENIN. C. Immunoblot analyses of RUNX2. D. mtDNA content was measured in HG-treated differentiated MC3T3-E1 cells. E. Immunoblot analyses of ACO2. F. Immunoblot analyses of CS. G. Immunoblot analyses of SUCLA2. H. Immunoblot analyses of SUCLG2. I. Immunoblot analyses of SUCLG1. J. Relative protein expression in percentage. All protein lysates for western blot were treated for four weeks in the presence of low or high glucose. Densitometry was measured using ImageJ software and normalized with loading control. Data was represented as % of loading control. Student t-test was used for data analysis. P <0.05 indicated significance. Abbreviations: OM = differentiated cells under low glucose conditions; OM, HG+ = differentiated cells under high glucose conditions. N = 6 per culture condition.
(TIF)

S1 Table Succinyl—CoA synthetase (SCS) activity in HG-treated osteoblasts. (XCF)

S1 Raw images (PDF)
