# Supplementary figures and images for: High glucose-induced inhibition of osteoblast like MC3T3-E1 differentiation promotes mitochondrial perturbations
Source: PLoS One. 2022 Jun 17;17(6):e0270001. doi: 10.1371/journal.pone.0270001 (PMC9205493; doi:10.1371/journal.pone.0270001)

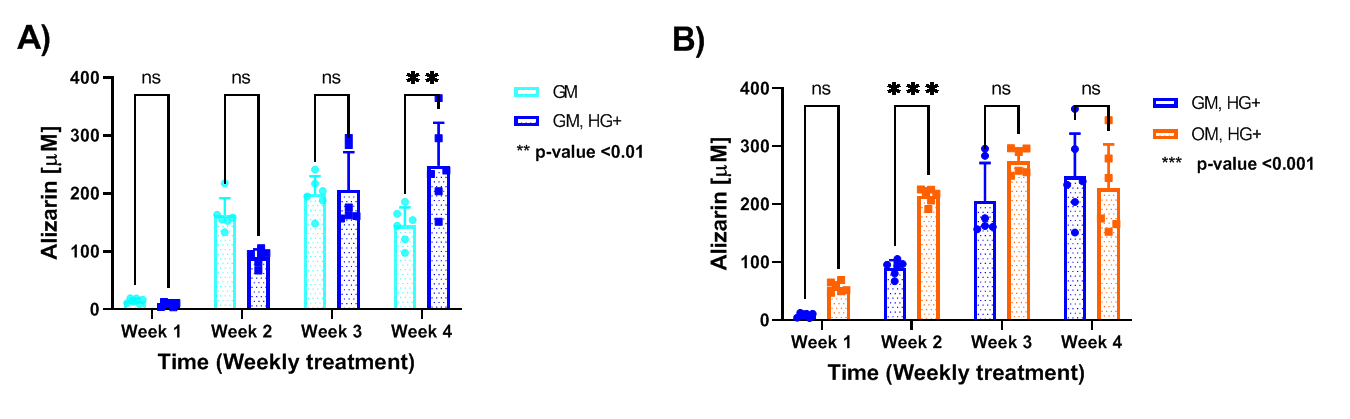

Supplement: S1 Fig — A. Quantitative analysis of Alizarin S red staining comparing undifferentiated MC3T3-E1 cells in the absence (GM)or presence of HG (GM, HG+). B. Quantitative analysis of Alizarin S red staining comparing undifferentiated (GM) and differentiated (OM) MC3T3 –E1 cells in the presence of HG (HG+). Assay were performed in triplicates at the end of each week for four weeks. Error bars represent standard deviation (SD). Abbreviations: GM = minimal growth media; OM = Osteogenic media; HG = high glucose, 30.5 mM final concentration. (TIF) [file pone.0270001.s001.tif]

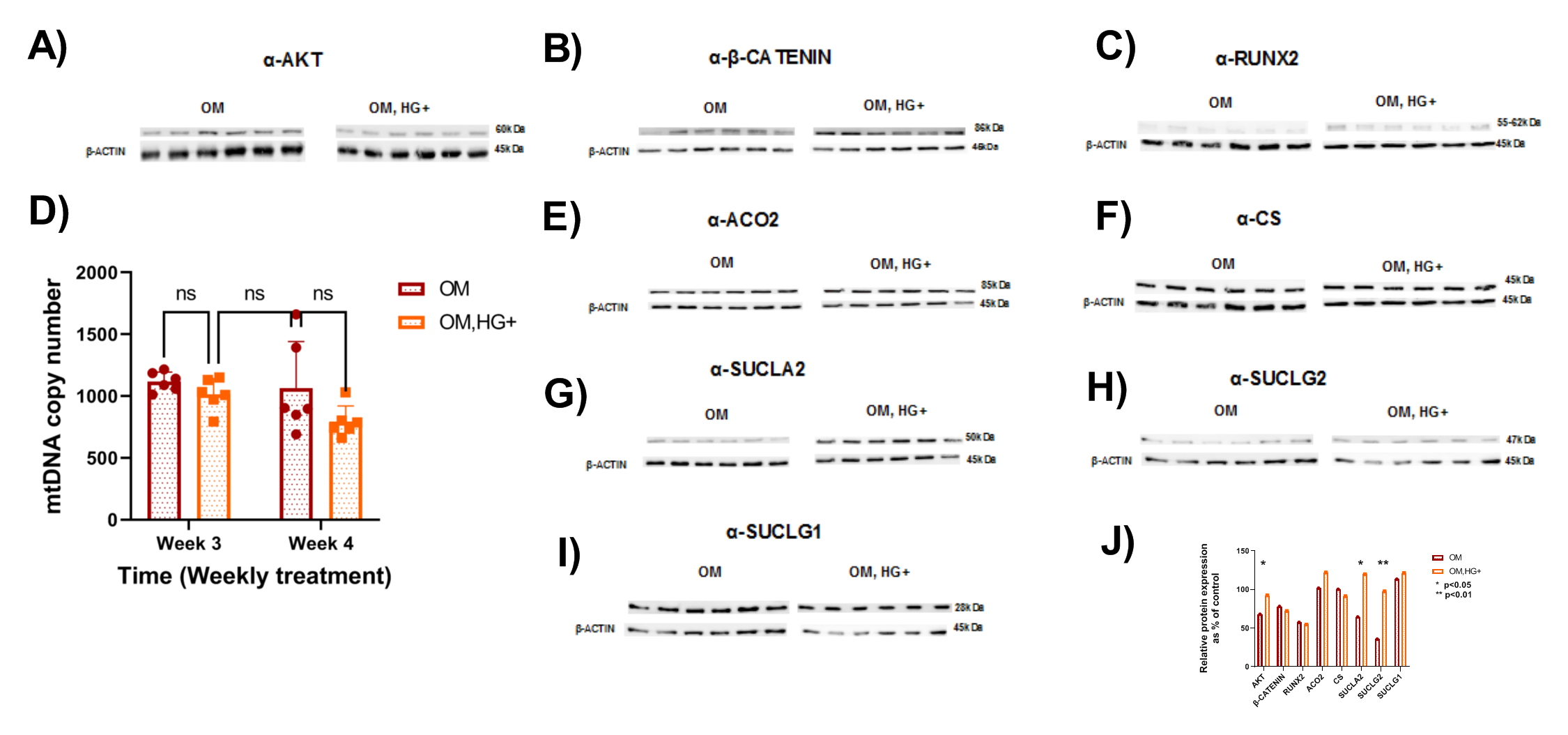

Supplement: S2 Fig — A. Immunoblot analyses of AKT. B. Immunoblot analyses of β-CATENIN. C. Immunoblot analyses of RUNX2. D. mtDNA content was measured in HG-treated differentiated MC3T3-E1 cells. E. Immunoblot analyses of ACO2. F. Immunoblot analyses of CS. G. Immunoblot analyses of SUCLA2. H. Immunoblot analyses of SUCLG2. I. Immunoblot analyses of SUCLG1. J. Relative protein expression in percentage. All protein lysates for western blot were treated for four weeks in the presence of low or high glucose. Densitometry was measured using ImageJ software and normalized with loading control. Data was represented as % of loading control. Student t-test was used for data analysis. P <0.05 indicated significance. Abbreviations: OM = differentiated cells under low glucose conditions; OM, HG+ = differentiated cells under high glucose conditions. N = 6 per culture condition. (TIF) [file pone.0270001.s002.tif]
