# Supplementary material for: High glucose-induced inhibition of osteoblast like MC3T3-E1 differentiation promotes mitochondrial perturbations
Source: PLoS One. 2022 Jun 17;17(6):e0270001. doi: 10.1371/journal.pone.0270001 (PMC9205493; doi:10.1371/journal.pone.0270001)
Supplement: S1 Raw images — (PDF) [file pone.0270001.s004.pdf]

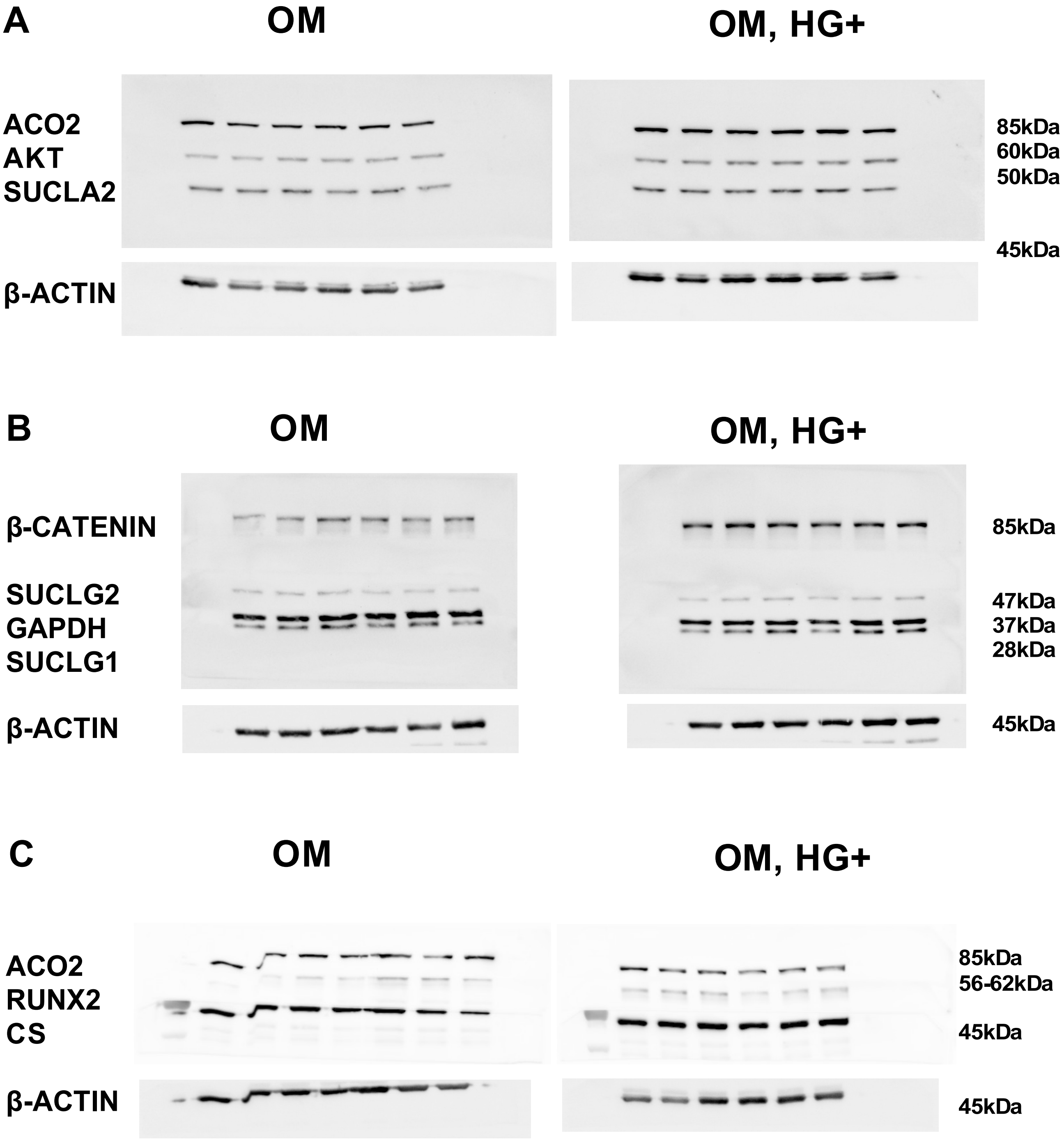
A
OM
OM, HG+
ACO2
AKT
SUCLA2
β-ACTIN
85kDa
60kDa
50kDa
45kDa
B
OM
OM, HG+
β-CATENIN
SUCLG2
GAPDH
SUCLG1
β-ACTIN
85kDa
47kDa
37kDa
28kDa
45kDa
C
OM
OM, HG+
ACO2
RUNX2
CS
β-ACTIN
85kDa
56-62kDa
45kDa
45kDa

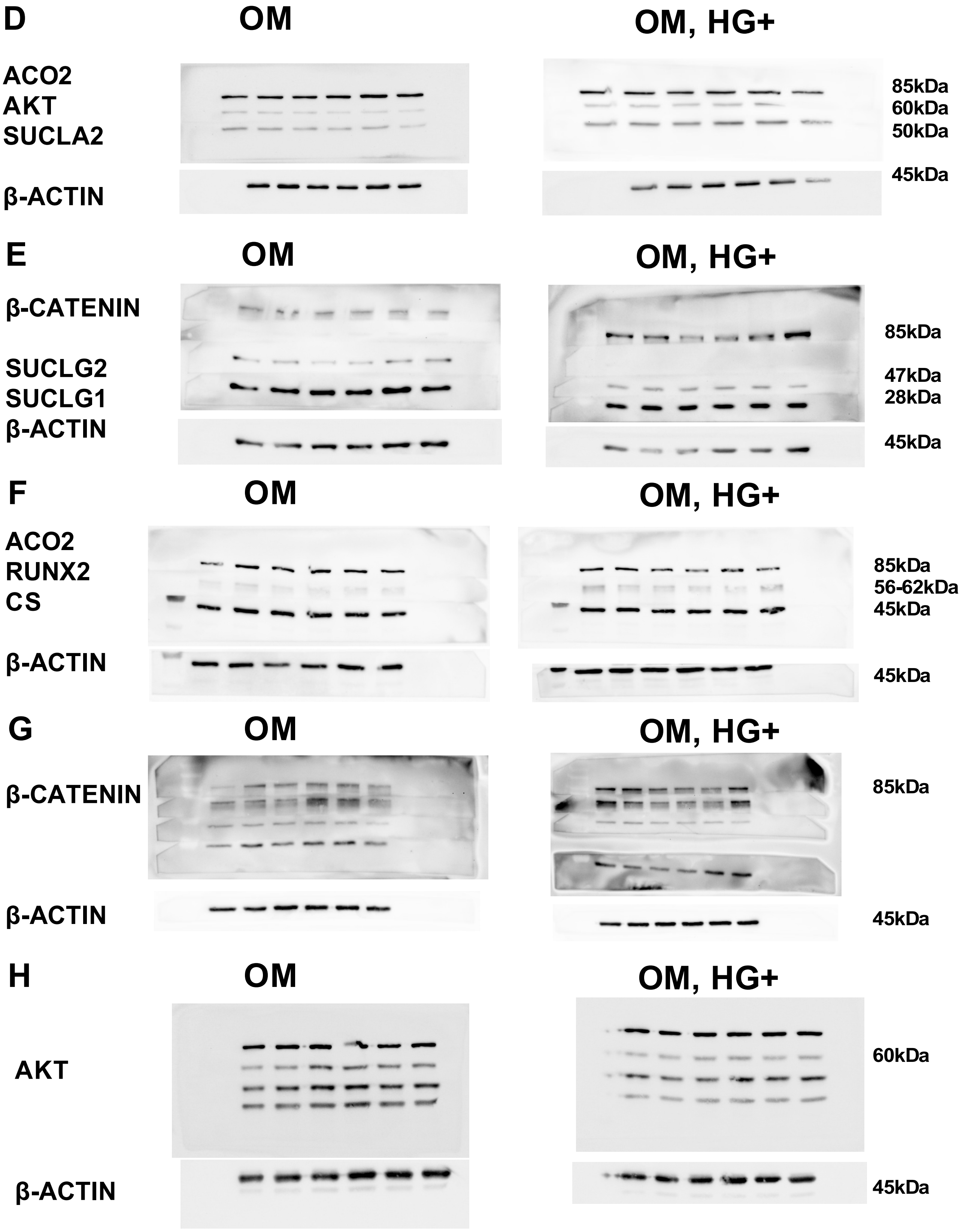
D
OM
OM, HG+
ACO2
AKT
SUCLA2
β-ACTIN
85kDa
60kDa
50kDa
45kDa
E
OM
OM, HG+
β-CATENIN
SUCLG2
SUCLG1
β-ACTIN
85kDa
47kDa
28kDa
45kDa
F
OM
OM, HG+
ACO2
RUNX2
CS
β-ACTIN
85kDa
56-62kDa
45kDa
45kDa
G
OM
OM, HG+
β-CATENIN
β-ACTIN
85kDa
45kDa
H
OM
OM, HG+
AKT
β-ACTIN
60kDa
45kDa

S1_Raw_Images.Raw Images used in Western Blot analysis. Three Immunoblots were used to detect expression of bone and mitochondrial proteins. Immunoblots from A to C correspond to detection of proteins expressed on Week 3 of differentiation and High Glucose treatment. Immunoblots from D to H correspond to detection of proteins expressed on Week 4 of differentiation and High Glucose treatment. Membranes were prepared as described in Methods. Kaleidoscope protein marker in the first lane (from left to right) was not detected by the ChemiDocMP Imaging System (Bio-Rad) and was used as reference for proper separation of the Membrane strips. A) Immunoblot analysis of ACO2, AKT, and SUCLA2 on week 3. B) Immunoblot analysis of β-CATENIN, SUCLG2, and SUCLG1 on week 3. C) Immunoblot analysis of RUNX2 and CS on week 3. D) Immunoblot analysis of ACO2, and SUCLA2 on week 4. AKT expression was not used for analysis. E) Immunoblot analysis of SUCLG2, and SUCLG1 on week 4. β-CATENIN expression was not used for analysis. F) Immunoblot analysis of RUNX2 and CS on week 4. G) Immunoblot analysis of β-CATENIN on week 4. H) Immunoblot analysis of AKT on week 4. β-actin was used as loading control. GAPDH was initially tested as loading control as seen in western blot B. Immunoblots were analyzed using ImageJ software. Unpaired t-test with Welch correction was used for statistical analysis. Sample size N=6, OM= Osteogenic media, OM, HG+= Osteogenic media in the presence of high glucose.
